# Eosinophilic granuloma: case report

**DOI:** 10.1186/1546-0096-12-S1-P298

**Published:** 2014-09-17

**Authors:** Angela Mauro, Carmen granato, Antonio Mellos, Grazia Cantelmi, Maria Francesca Gicchino, Federica Messa, Alma Nunzia Olivieri

**Affiliations:** 1 Pediatric, Second University of Naples, Naples, Italy

## Introduction

Lameness is a symptom very common in childhood, it depends on inflammatory, neoplastic, infective, orthopedic diseases.

## Objectives

To describe a clinical report of a Eosinophilic Granuloma beginning with lameness.

## Methods

V. 29 months old comes to our hospital because of persistent lameness . The symptoms began three months before, after a trauma. For persisting pain and lameness the child was checked by an orthopaedist who diagnosed “hip transitory synovitis”. Infact radiography and ultrasonography of the hip and femoral head were negative. The child was prescribed therapy with NSAIDs. At first the child’s conditions improved, but after sometime his leg was in pain again. When he came to our observation in the articular examinations revealed pain, functional limitation of the right leg and lameness, inflammatory index(ERS and CRP) were normal. Suspected of having a Perthes disease the child underwent to another right leg radiography that revealed the presence of a remodelling area with an osteolytic lesion near the big trochanter. The CT scan confirmed the osteolytic lesion with sclerotic regular margins eroding the cortical bone. Scintigrapy performed with tc99 showed solitary bone lesion with a slightly increased metabolism. So we decided to performed a needle biopsy to define the diagnosis.

## Results

Histologic examination of the lesion revealed connective proliferation, edema, with different subtypes of immune cells ( lymphocytes, granulocytes, eosinophils) and histiocytes . so was made diagnosis of Eosinophilic Granuloma and was performed a surgical curettage of the lesion.

## Conclusion

Eosinophilic granuloma (EG) is a rare histiocytic disorder due to clonal proliferation of Langerhans cells. It is characterized by single or multiple bone lesions involving cranium, mandible,pelvis, ribs, spine and long bones , in particular femur. Eosinophilic granuloma onsets with pain, getting worse during the night, and edema, in this phase laboratory tests are normal. Imaging ( radiography, CT scan, scintigrapy,magnetic resonance) shows single or multiple osteolytic lesions, with cortical erosion. This disease has to be differentiated by a destructive lesion includes malignancy such as Ewing sarcoma, lymphoma, leukemia, and metastasis as well as osteomyelitis. Biopsy of one of the lesions is often needed for diagnosis.

## Disclosure of interest

None declared
